# Identification of a Glycosyltransferase Signature for Predicting Prognosis and Immune Microenvironment in Neuroblastoma

**DOI:** 10.3389/fcell.2021.769580

**Published:** 2022-01-06

**Authors:** Yongliang Sha, Lei Han, Bei Sun, Qiang Zhao

**Affiliations:** ^1^ Department of Pediatric Oncology, Tianjin Medical University Cancer Institute and Hospital, Tianjin, China; ^2^ National Clinical Research Center for Cancer, Tianjin Medical University Cancer Institute and Hospital, Tianjin, China; ^3^ Key Laboratory of Cancer Prevention and Therapy, Tianjin, China; ^4^ Tianjin’s Clinical Research Center for Cancer, Tianjin, China; ^5^ Cancer Molecular Diagnostics Core, Tianjin Medical University Cancer Institute and Hospital, Tianjin, China; ^6^ Department of Outpatient Office, Tianjin Medical University Cancer Institute and Hospital, Tianjin, China

**Keywords:** GTscore, neuroblastoma, glycosyltransferase, prognosis, ganglioside GD2, immunotherapy, bioinformatics analysis 3/32

## Abstract

Neuroblastoma (NB) is one of the most common solid tumors in children. Glycosyltransferases (GTs) play a crucial role in tumor development and immune escape and have been used as prognostic biomarkers in various tumors. However, the biological functions and prognostic significance of GTs in NB remain poorly understood. The expression data from Gene Expression Omnibus (GEO) and Therapeutically Applicable Research to Generate Effective Treatments (TARGET) were collected as training and testing data. Based on a progression status, differentially expressed GTs were identified. We constructed a GTscore through support vector machine, least absolute shrinkage and selection operator, and Cox regression in NB, which included four prognostic GTs and was an independent prognostic risk factor for NB. Patients in the high GTscore group had an older age, MYCN amplification, advanced International Neuroblastoma Staging System stage, and high risk. Samples with high GTscores revealed high disialoganglioside (GD2) and neuron-specific enolase expression levels. In addition, a lack of immune cell infiltration was observed in the high GTscore group. This GTscore was also associated with the expression of chemokines (CCL2, CXCL9, and CXCL10) and immune checkpoint genes (cytotoxic T-lymphocyte–associated protein 4, granzyme H*,* and granzyme K). A low GTscore was also linked to an enhanced response to anti–PD-1 immunotherapy in melanoma patients, and one type of tumor was also derived from neuroectodermal cells such as NB. In conclusion, the constructed GTscore revealed the relationship between GT expression and the NB outcome, GD2 phenotype, and immune infiltration and provided novel clues for the prediction of prognosis and immunotherapy response in NB.

## Introduction

Neuroblastoma (NB) is a malignant childhood solid tumor of neuroectodermal cells derived from the sympathetic nervous system and the primary cause of pediatric cancer-related mortality (Maris et al., 2007). Despite advances in multimodality therapy, the prognosis of high-risk NB patients remains poor. Age, tumor stage, mitosis-karyorrhexis index (MKI), MYCN amplification, and ALK expression are used to predict the clinical outcomes of NB (Perel et al., 2004; Chang et al., 2020; Sokol et al., 2020). However, there are limitations in the accuracy and specificity of each index. Therefore, it is urgent to explore new prognostic markers.

Glycosyltransferases (GTs) are a series of enzymes that catalyze the formation of glycosidic linkages to diverse acceptor molecules, such as proteins, lipids, hormones, and oligosaccharides. The altered expression of GTs leading to aberrant glycosylation patterns has been found in various cancers and is considered as a biomarker of cancer, such as small cell lung cancer (Yoshida et al., 2001), NB (Berois et al., 2013), melanoma (Hu et al., 2019), and triple-negative breast cancer (TNBC) (Narvaez et al., 2020). Moreover, the glycosylation products catalyzed by various GTs also play an important role in the biological function of cancer cells, including proliferation, migration, metastasis, immune escape, and apoptosis (Cavdarli et al., 2019). A large number of studies have been performed to investigate the functions of GTs in various types of tumors (Huang et al., 2015; Schultz et al., 2016; Dong et al., 2017; Bhat et al., 2018). Unfortunately, to date, there is still a lack of an in-depth analysis of the role of these GTs in NB. With large amounts of molecular sequence data accumulating gradually, bioinformatics based on the massive microarray datasets provides a promising strategy for these studies.

Disialoganglioside (GD2), which is synthesized by GTs, is highly expressed on NB tumor cells, with absent or weak expression in normal tissues (Balis et al., 2020). In addition to NB, GD2 is also overexpressed in other neuroectodermal tumors and sarcomas, such as melanoma (Hamilton et al., 1993), brain tumors (Mennel et al., 2000), osteosarcoma (Poon et al., 2015), and Ewing sarcoma (Fisher et al., 2016), as well as several types of breast cancer (Orsi et al., 2017) and small cell lung cancer (Cheresh et al., 1986). GD2 has many biological functions, involving tumor cell proliferation, migration, invasion, and apoptosis (Liu et al., 2014; Mansoori et al., 2019). Schulz et al. showed that soluble GD2 (sGD2) levels in NB patients were associated with disease progression (Schulz et al., 1984). Furthermore, tumor-derived GD2 inhibits T-cell proliferation (Ladisch et al., 1992), and therefore may promote tumor immune evasion. Given its high prevalence in NB tissues compared with normal tissues, GD2 is used as a target for cancer immunotherapy. As GD2-positive tumors are more responsive to such targeted therapy, GD2 has been recommended as a therapeutic monitoring tool in NB patients treated with GD2-targeted immunotherapy (Terzic et al., 2018). Neuron-specific enolase (NSE) is currently considered a useful marker in the diagnosis, prognosis, and follow-up of related neuroendocrine tumors (Zeltzer et al., 1983; Shine et al., 1990; Giovanella et al., 1995). In view of the biological importance of GD2 and NSE, it is imperative to detect the expression levels of GD2 and NSE in cancer cells.

In this study, the expression profiles of GT genes in the Gene Expression Omnibus (GEO) database (GSE49710 dataset) were downloaded and analyzed to identify differentially expressed GTs in NB. Gene ontology (GO) and Kyoto Encyclopedia of Genes and Genomes (KEGG) pathway analyses revealed that genes were mainly involved in the glycosphingolipid biosynthetic process. We systemically assessed the identified differentially expressed GTs, especially the genes involved in the GD2 synthesis pathway. Then, using the least absolute shrinkage and selection operator (LASSO), Cox, and support vector machine (SVM) algorithm, we developed a GTscore for predicting the prognosis of NB patients *via* the GSE49710 dataset and verified the feasibility of the GTscore through the Therapeutically Applicable Research to Generate Effective Treatments (TARGET) dataset. The GTscore has a relation with clinicopathological risk factors and could be used as an independent risk factor. Furthermore, our GTscore correlated with immune cell infiltration, GD2 molecular phenotypes, and NSE expression levels. Importantly, the GTscore could accurately predict the response to immunotherapy in melanoma, another type of tumor derived from neuroectodermal cells. In summary, we built a 4-GT signature model that could be used as a predictor of NB survival, responsiveness to immunotherapy in melanoma, and GD2 molecular phenotypes of tumor cells.

## Materials and Methods

### Data Sources and Data Preprocessing

The gene expression data and clinical information of GSE49710 (Zhang et al., 2015) were downloaded from the GEO database (https://www.ncbi.nlm.nih.gov/geo/). A total of 498 NB samples including RNA-seq and clinical information (sex, age, International Neuroblastoma Staging System [INSS] stage, MYCN amplification status, and progression) were used as the training set. The GSE112447, GSE90689, and GSE142293 datasets were also downloaded for GD2 molecular phenotype analysis. Probes were converted to corresponding gene symbols according to the “clusterProfiler” R package (Yu et al., 2012) with the platforms GPL16876 and GPL6480 to obtain a gene expression matrix. The gene expression profile and clinical data (sex, age, INSS stage, MYCN amplification status, MKI, grade, COG risk, relapse, and progression) of TARGET-NB were downloaded from the TARGET database (154 patients) (https://ocg.cancer.gov/programs/target), which were used for validation of differentially expressed genes. Gene sequencing data and immunotherapy information of The Cancer Genome Atlas Skin Cutaneous Melanoma (TCGA-SKCM) were downloaded from TCGA database (https://portal.gdc.cancer.gov/). Ensembl IDs were converted to corresponding gene symbols with the clusterProfiler R package. Then, these gene expression matrices were quantile standardized and log2 transformed for subsequent analysis.

### Screening Differentially Expressed GTs

The processed gene expression matrix of GSE49710 was subjected to differentially expressed GT gene (DEG) analysis to screen genes that were differentially expressed between the progression and nonprogression groups. The “limma” (Ritchie et al., 2015) R package was utilized, and genes were identified as significant DEGs if |log2FC| value >0.5 and false discovery rate (FDR) <0.05. Then, univariable Cox regression analysis was applied to identify DEGs related to the prognosis of NB patients.

### Functional Enrichment Analysis

In this study, the clusterProfiler package was used to complete the enrichment analysis with DEGs, aiming to provide a primary understanding of the biological function of these genes. *p* < 0.05 and FDR < 25% were identified as significant.

### Protein-Protein Interaction Network Construction

The STRING (version 11.0, https://string-db.org) database was used to construct protein–protein interaction (PPI) network (Szklarczyk et al., 2015). Then, the network was visualized *via* Cytoscape software (version 3.5.1).

### Feature Selection and Identification of GTscore

The LASSO is a shrinkage estimation method whose basic premise is to minimize the residual sum of squares under the constraint by restricting coefficients of some features to zero and further obtain shrinkage subsets (Tibshirani, 1997). In this study, a LASSO Cox regression model was applied to identify DEGs associated with progression-free survival (PFS). Tenfold cross-validation for tuning parameter selection was performed, and the partial likelihood deviance met the minimum criteria with the “glmnet” R package.

The SVM method is used to find the best variable combination by subtracting the inputted feature vector (Huang et al., 2018). In the current study, the gene expression matrix of 498 pediatric NB samples was subjected to SVM operation and randomly divided into a training group and verification group with the “glmnet” and “e1071” R packages. The ganglioside synthesis–related DEGs were combined in various combinations to establish the optimal predictive classification model, and the smallest classification error and highest accuracy of the prediction model of the training group were externally verified to obtain the optimal combination.

The advantage of these algorithms is that they can retain subset shrinkage, so they are more suitable for processing multicollinearity data. We then obtained the coefficients of screened DEGs to construct a GTscore. The risk score for the individual samples was calculated as follows:

GTscore = β1∗X1+β2∗X2+…βi∗Xi …+ βn∗Xn

where β_i_ represents the correlation coefficient for gene *i*, and *X*_*i*_ represents the expression of gene *i*.

According to the median cutoff value of the GTscore, the patients were divided into high and low GTscore groups. The log-rank test was applied to compare the overall survival (OS), event-free survival (EFS), and recurrence-free survival (RFS) differences between the two groups. In addition, univariate Cox regression and multivariable Cox regression were used to assess the independent prognostic value of the GTscore.

### Immune Cell Infiltration Evaluation

It is generally believed that the degree of immune cell infiltration is associated with tumor occurrence, development, treatment, and clinical prognosis (Whiteside, 2008; Asgharzadeh et al., 2012; Wienke et al., 2021). Studies have found that GT genes are related to tumor immune escape (Wang et al., 2019; Liu et al., 2020). Accordingly, we explored the relationship between the GTscore and immune cell infiltration. With previously published marker gene sets representing diverse immune cell types (Bindea et al., 2013), the relative abundance of immune cell types for a single sample was quantified using the R package “GSVA” (Hanzelmann et al., 2013) and single-sample gene set enrichment analysis (ssGSEA) function under standard settings. To further explore the inflammatory status, we estimated the expression levels of a series of chemokines in the high and low GTscore groups. In addition, the relationships between the expression levels of immune checkpoint genes such as cytotoxic T-lymphocyte–associated protein 4 (CTLA-4) and granzymes H and K (GZMH, GZMK) and the GTscore were evaluated.

### Correlation of the GTscore With GD2 Molecular Phenotypes and NSE Expression Levels

GD2 and NSE were the significant markers for neuroendocrine tumors, including NB, melanoma, and small cell lung cancer. Because of the importance of GD2 and NSE, we assessed the correlation between GTscore and GD2 molecular phenotypes and NSE expression levels.

To evaluate the relationship between GTscore and GD2 molecular phenotypes, we compared the GTscore between putatively GD2 (+) (GSE112447) and GD2 (−) NB samples (GSE90689), GD2 (++) cell lines, and GD2 (+/−) cell lines (GSE142293) (Sorokin et al., 2020), GD2 (−) acute lymphoblastic leukemia (ALL) (Macher et al., 1982; Pick et al., 1986) and GD2 (+) NB (Wu et al., 1986; Terzic et al., 2018) (TARGET database), GD2 (−) non-TNBC, and GD2 (+) TNBC samples (Orsi et al., 2017) (TCGA database).

Furthermore, Spearman correlation analysis was used to analyze the relationship between GTscore and NSE expression levels in NB (Zhang et al., 2015) (GSE49710), melanoma (Bakhoum and Esmaeli, 2019) (TCGA database), and small cell lung cancer (TCGA database).

### Estimation of Immunotherapeutic Response

Tumor immunotherapy using immune checkpoint inhibitors (ICIs) is a promising treatment for solid tumors. GTs play an important role in cancer cell immune escape (Galvan et al., 2000; Wang et al., 2019). Thus, we explored the correlation between GTscore and tumor response to ICIs in SKCM, a type of neuroendocrine tumor. Receiver operating characteristic (ROC) curves were applied to investigate the predictive ability of the GTscore for immunotherapeutic response. The Kaplan–Meier method was used to analyze 1- and 3-year PFS stratified by GTscore.

### Statistical Analysis

Student *t* test, analysis of variance, Mann–Whitney Wilcoxon test, and Kruskal–Wallis test were used to compare continuous variables and ordered categorical variables. Pearson *χ* ^2^ test and Fisher exact test were used to compare unordered categorical variables. Survival curves were constructed using the Kaplan–Meier method and were compared using the log-rank test and “survminer” R package. The optimal cutoff point for continuous variables was determined by the “surv_cutpoint” function in the survminer R package. A Cox proportional hazard regression model was performed to calculate hazard ratios (HRs) and 95% confidence intervals (CIs). Multiple testing corrections were performed using the Benjamini–Hochberg procedure and produced FDRs. All the tests were two-sided, and *p* < 0.05 or FDR < 25% was regarded as indicating statistical significance unless otherwise stated. All analyses were performed using R package version 3.4.2 (https://cran.r-project.org/).

## Results

### Identification of Differentially GT Expressed Genes Associated With NB Progression and Patient Survival

**FIGURE 1:**
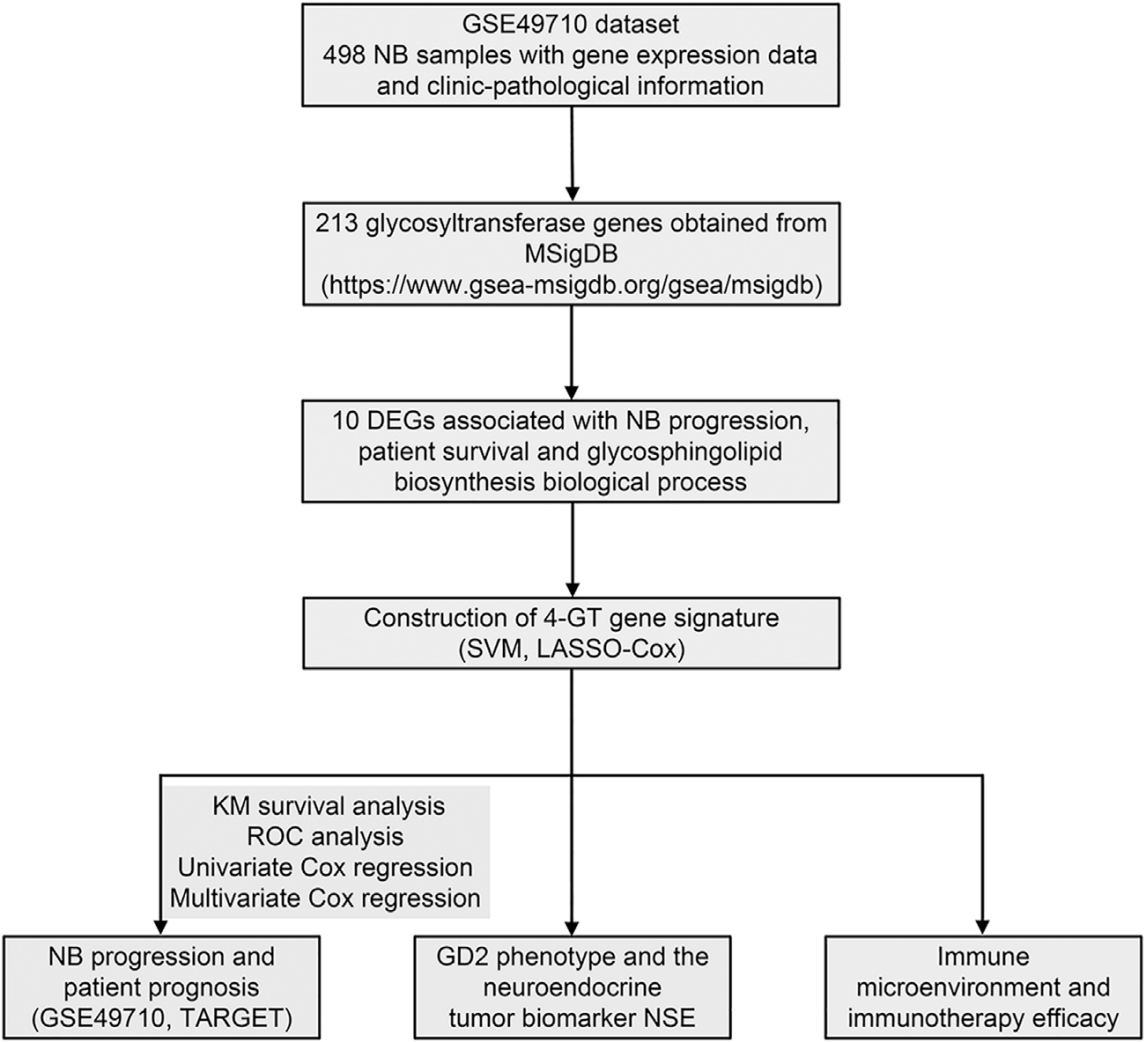
Flowchart of this study.

**FIGURE 2:**
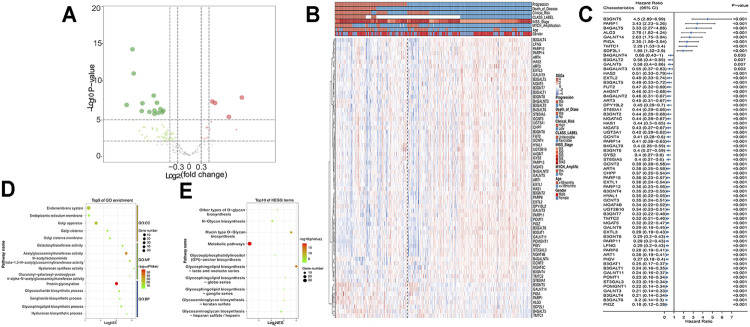
DEGs in GSE49710 dataset. **(A)** Volcano plot shows DEGs between progression and nonprogression NB samples. Red points represent upregulated DEGs. Green points denote downregulated DEGs. Gray points denote genes with no significant differences. **(B)** Heat map showing the association between 64 DEGs and clinical characteristics annotated on the top. **(C)** Forest plot of univariate Cox regression results of DEGs. GO **(D)** and KEGG **(E)** analyses of DEGs.

The workflow is shown in Figure 1. First, 213 GT genes were collected from MSigDB (Molecular Signatures Database) (https://www.gsea-msigdb.org/gsea/msigdb). We then explored which genes were differentially expressed between progression and nonprogression NB samples based on the dataset GSE49710, the cohort of 498 NB samples with gene expression data, and clinicopathological information (Table 1). As shown in Figures 2A–C, we found that 64 GT genes were significantly related to NB progression and survival. GO analysis results showed that DEGs were mostly enriched in glycosphingolipid biosynthetic process, protein glycosylation, and galactosyltransferase activity (Figure 2D). KEGG analysis results suggested that these DEGs were mainly involved in glycosphingolipid biosynthesis and glycan biosynthesis (Figure 2E). The results above suggested that the glycosphingolipid biosynthesis biological process played an important role in the progression of NB. Anti-GD2 immunotherapy showed favorable clinical efficacy for high-risk NB (Rovigatti, 2020). GD2 is synthesized under the catalysis of GTs (Groux-Degroote et al., 2017), which are part of the DEGs. Thus, we focused on the 10 GD2 synthesis–related GT genes in subsequent analysis.

**TABLE 1:** Clinical pathological parameters of NB patients in the training set.

Features	Number (%)
Sex	
Female	211 (42.4%)
Male	287 (57.6%)
Age	
≤18 months	300 (60.2%)
>18 months	198 (39.8%)
MYCN amplification	
No	401 (80.5%)
Unknown	5 (1.0%)
Yes	92 (18.5%)
INSS stage	
St1	121 (24.3%)
St2	78 (15.7%)
St3	63 (12.7%)
St4	183 (36.7%)
St4S	53 (10.6%)
Clinical risk	
High	176 (35.3%)
Low	322 (64.7%)
Class label	
Favorable	181 (36.3%)
Unfavorable	91 (18.3%)
Unknown	226 (45.4%)
Progression	
No	315 (63.3%)
Yes	183 (36.7%)
Death from disease	
No	393 (78.9%)
Yes	105 (21.1%)

### Construction and Validation of GT Gene Prognostic Model

**FIGURE 3:**
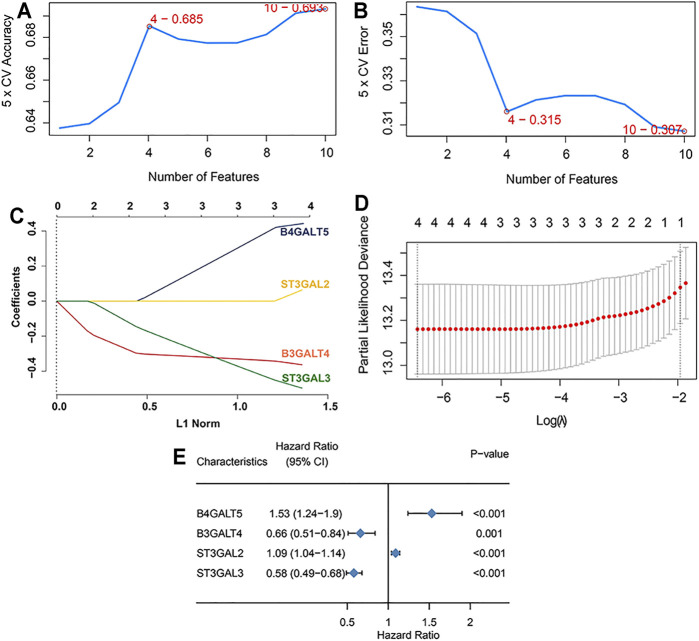
Construction of GTscore with machine learning in the training set. The accuracy **(A)** and the error **(B)** of the estimate generation for the SVM-RFE algorithm. **(C)** Ten-time cross-validation was performed for tuning parameter selection through the LASSO regression model. **(D)** LASSO coefficient profiles of prognostic GTs. **(E)** Forest plot showing the HR with 95% CI of prognostic GTs.

Difference analysis and survival analysis illustrated that these GD2 synthesis–related GT genes were significantly related to EFS (Supplementary Figure S1) and OS (Supplementary Figure S2). B4GALT5 and ST3GAL2 were considered as risk factors, whereas the remaining eight genes acted as protective factors. The PPI network and expression correlation analysis showed that they were highly intercorrelated (Supplementary Figure S3). With the SVM algorithm, we screened out the optimal gene combination: ST3GAL2, ST3GAL3, B4GALT5, and B3GALT4 (Figures 3A,B). Next, we conducted LASSO Cox regression analysis and obtained coefficients for each gene (Figures 3C–E). The final GTscore formula was as follows: GTscore = (0.4423) * B4GALT5 expression value + (−0.3630) * B3GALT4 expression value + (0.0636) * ST3GAL2 expression value + (−0.4962) * ST3GAL3 expression value. The ROC curve analyses revealed that the area under the curve (AUC) values at 1-, 3-, and 5-year EFS and OS for the GTscore were higher than those for individual genes (Supplementary Figure S4), indicating that the prognostic role of the GTscore was more accurate than that of a single gene alone.

**FIGURE 4:**
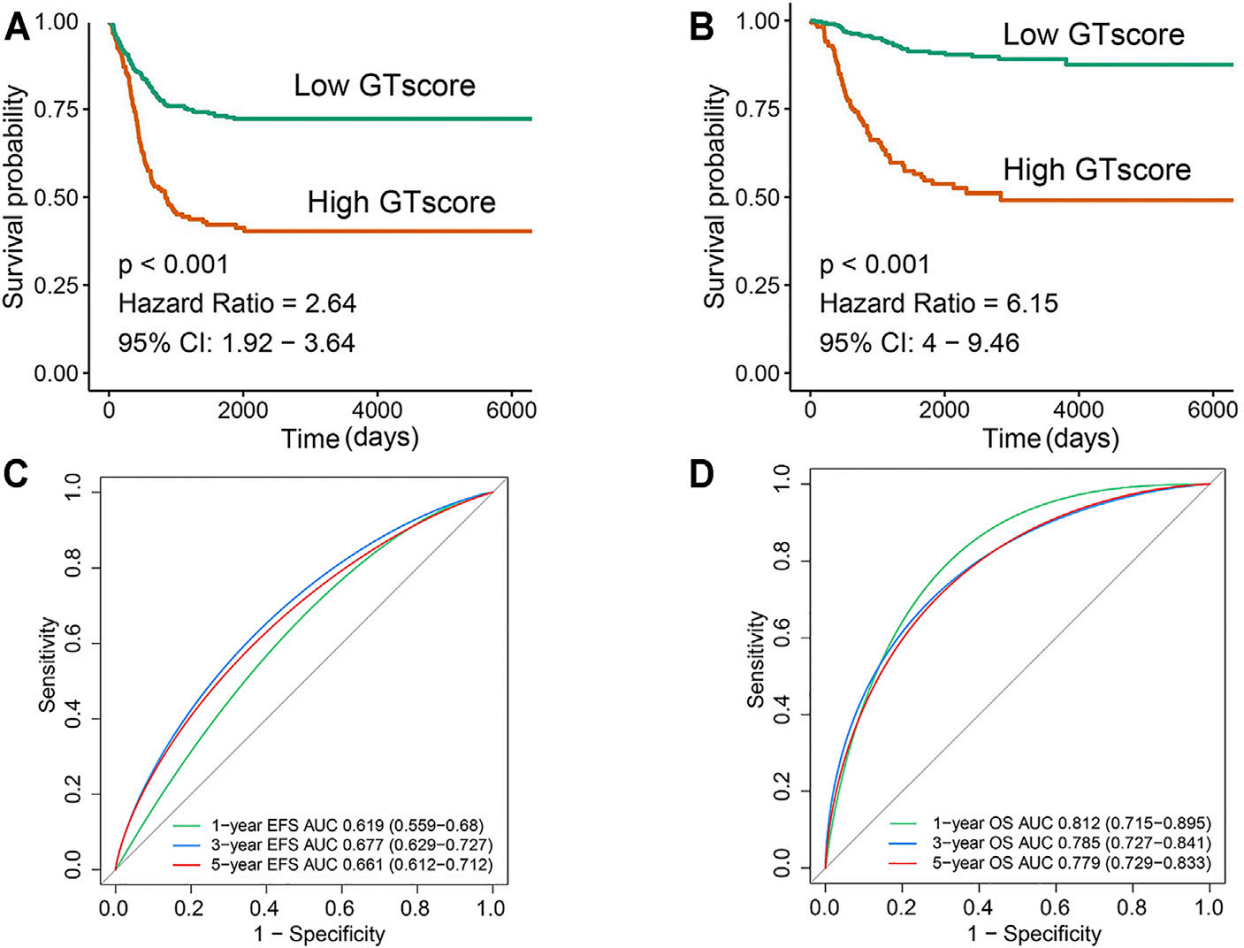
Validation of the prognostic value of the GTscore for NB patients in the training set. Kaplan–Meier analysis for EFS **(A)** and OS **(B)** of NB patients based on the risk stratification. ROC analysis for 1-, 3-, and 5-year EFS **(C)** and OS **(D)** prediction in NB patients. The AUCs at 1, 3, and 5 years were calculated to assess the predictive accuracy.

**FIGURE 5:**
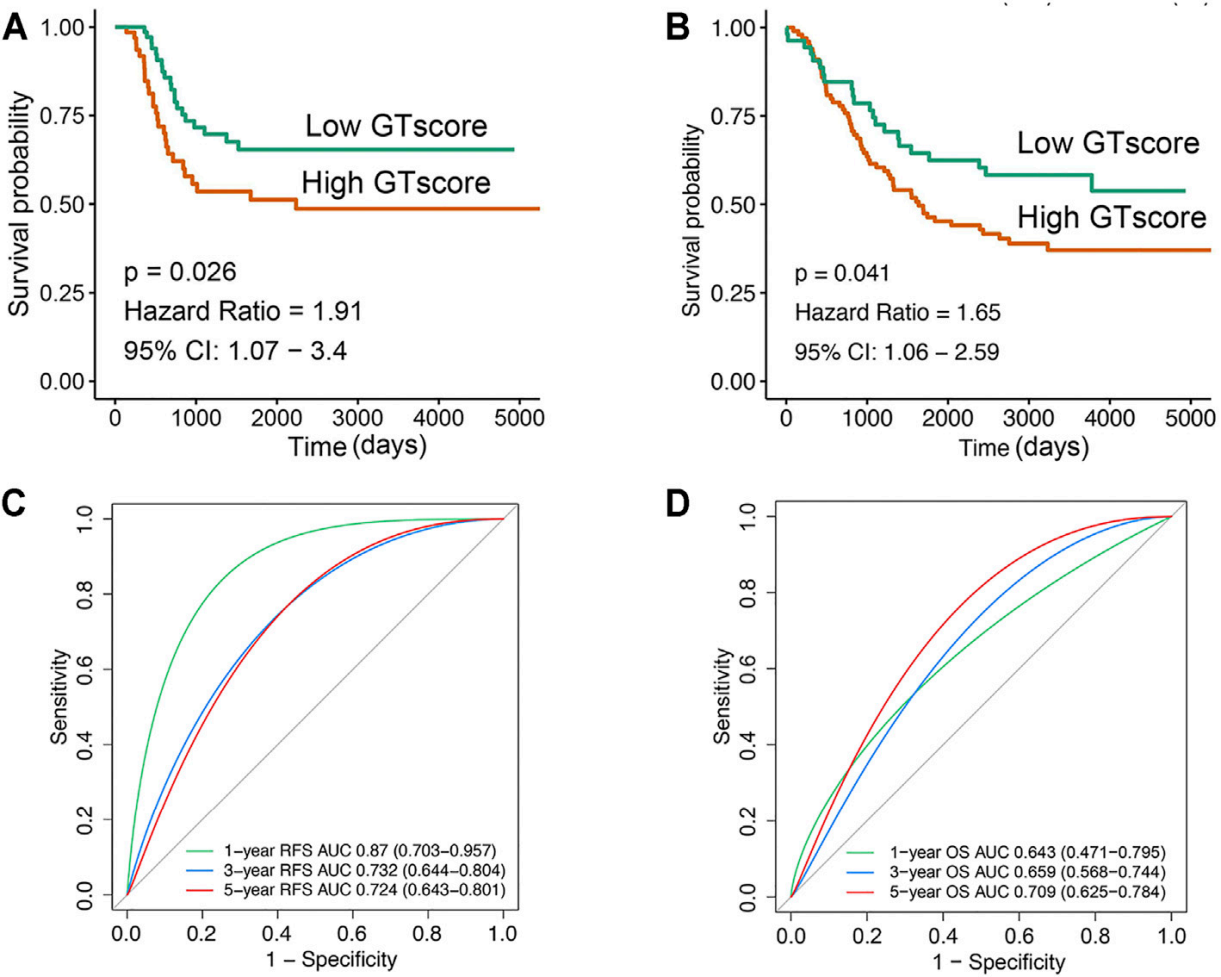
Validation of the prognostic value of GTscore for NB patients in the validation set. Kaplan–Meier analysis for RFS **(A)** and OS **(B)** of NB patients. ROC analysis for 1-, 3-, and 5-year RFS **(C)** and OS **(D)** prediction in NB patients. The AUCs at 1, 3, and 5 years were calculated to assess the predictive accuracy.

In the training cohort, Kaplan–Meier survival analysis results showed that the EFS and OS of patients in the high GTscore group were significantly lower than those in the low GTscore group (HR = 2.64, *p* < 0.001 for EFS, and HR = 6.15, *p* < 0.001 for OS) (Figures 4A, B). The AUCs of the GTscore to distinguish 1-, 3-, and 5-year EFS were 0.619, 0.677, and 0.661, respectively (Figure 4C), and the AUCs to distinguish 1-, 3-, and 5-year OS were 0.812, 0.785, and 0.779, respectively (Figure 4D), demonstrating great efficacy in distinguishing the prognostic status of NB. A summary of the clinical characteristics of the validation cohort is shown in Table 2. In the validation cohort, Kaplan–Meier survival analysis results showed that the RFS and OS of patients in the high GTscore group were significantly lower than those in the low GTscore group (HR = 1.91, *p* = 0.026 for RFS, and HR = 1.65, *p* = 0.041 for OS) (Figures 5A, B). The AUCs to distinguish 1-, 3-, and 5-year RFS were 0.870, 0.732, and 0.724 (Figure 5C), and 0.643, 0.659, and 0.709 for 1-, 3-, and 5-year OS (Figure 5D), suggesting that the GTscore was a robust prognostic model and had good predictive performance in different circumstances.

**TABLE 2:** Clinical pathological parameters of NB patients in the validation set.

Features	Number
Sex	
Female	64 (41.6%)
Male	90 (58.4%)
Age	
≤18 months	29 (18.8%)
>18 months	125 (81.2%)
Race	
Asian	1 (0.6%)
Black or African American	26 (16.9%)
Native Hawaiian or other pacific islander	2 (1.3%)
Unknown	13 (8.4%)
White	112 (72.7%)
MYCN amplification	
No	120 (77.9%)
Unknown	1 (0.6%)
Yes	33 (21.4%)
INSS stage	
St2b	1 (0.6%)
St3	6 (3.9%)
St4	126 (81.8%)
St4s	21 (13.6%)
Hyperdiploid	
No	65 (42.2%)
Unknown	1 (0.6%)
Yes	88 (57.1%)
Histology	
Favorable	28 (18.2%)
Unfavorable	115 (74.7%)
Unknown	11 (7.1%)
Grade	
Differentiating	10 (6.5%)
Undifferentiated	120 (77.9%)
Unknown	24 (15.6%)
MKI	
High	33 (21.4%)
Intermediate	42 (27.3%)
Low	48 (31.2%)
Unknown	31 (20.1%)
COG risk	
High	127 (82.5%)
Intermediate	13 (8.4%)
Low	14 (9.1%)
Progression	
No	88 (57.1%)
Yes	66 (42.9%)
Relapse	
No	107 (69.5%)
Yes	47 (30.5%)

### The GTscore Is an Independent Prognostic Factor

**FIGURE 6:**
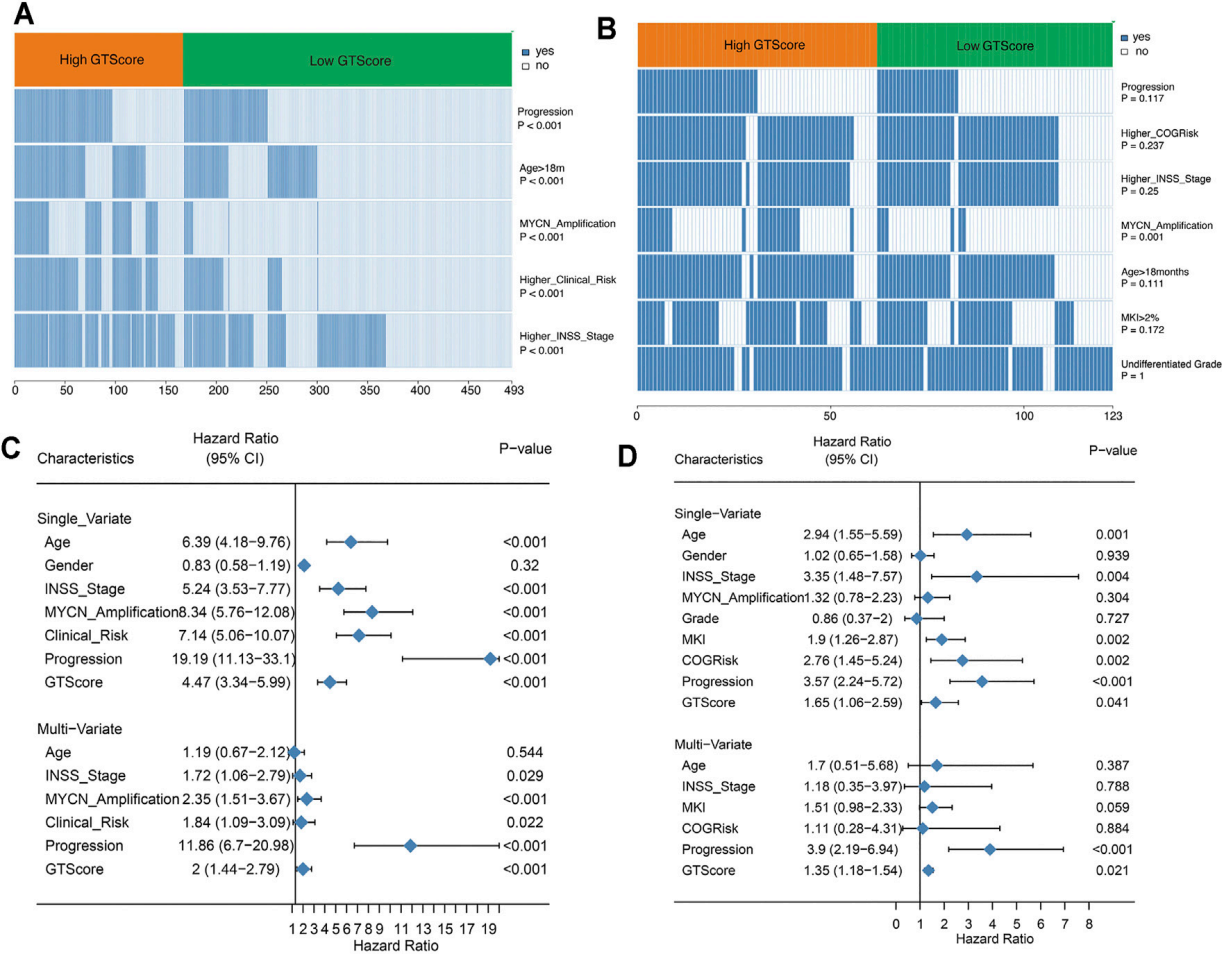
GTscore is related to the clinical characteristics of NB and is an independent prognostic factor. The correlation heat map showing the association of GTscore with clinical characteristics in the training set **(A)** and validation set **(B)**. Univariate analysis and multivariate analysis of the GTscore, age, sex, INSS stage, MYCN amplification, clinical risk, disease progression, MKI, and OS in the training set **(C)** and validation set **(D)**.

Currently, clinicopathological factors are still the most important guidelines and are used to predict the prognosis of NB. Therefore, we explored the correlation between GTscore and clinical characteristics. Correlation analysis showed that GTscore was significantly related to age, INSS staging, MYCN amplification, high risk, and disease progression in the training cohort (Figure 6A, Supplementary Figure S5A), and MYCN amplification in the validation cohort (Figure 6B, Supplementary Figure S5B) (all *p* < 0.05).

In the training cohort, univariate Cox regression results showed that patients with older age (>18 months), higher INSS stage, MYCN amplification, higher clinical risk, disease progression, and higher GTscore had a worse prognosis (all *p* < 0.05) (Figure 6C). Subsequently, the multivariate Cox regression analysis showed that high INSS staging, MYCN amplification, high clinical risk, disease progression, and high GTscore (*p* < 0.001) were independent risk factors for NB patients (Figure 6C).

In the validation cohort, the correlation analysis showed that patients with high GTscore were older (>18 months), had higher INSS stage and MYCN amplification, had higher MKI (>4%), and had higher clinical risk and disease progression (all *p* < 0.05) (Figure 6D). Subsequently, these significant indicators were further included in the multivariate Cox regression analysis, and the results showed that disease progression (HR = 3.90, *p* < 0.001) and high GTscore (HR = 1.35, *p* = 0.021) were independent risk factors for NB patients (Figure 6D).

### Correlation of the GTscore With Immune Cell Infiltration

**FIGURE 7:**
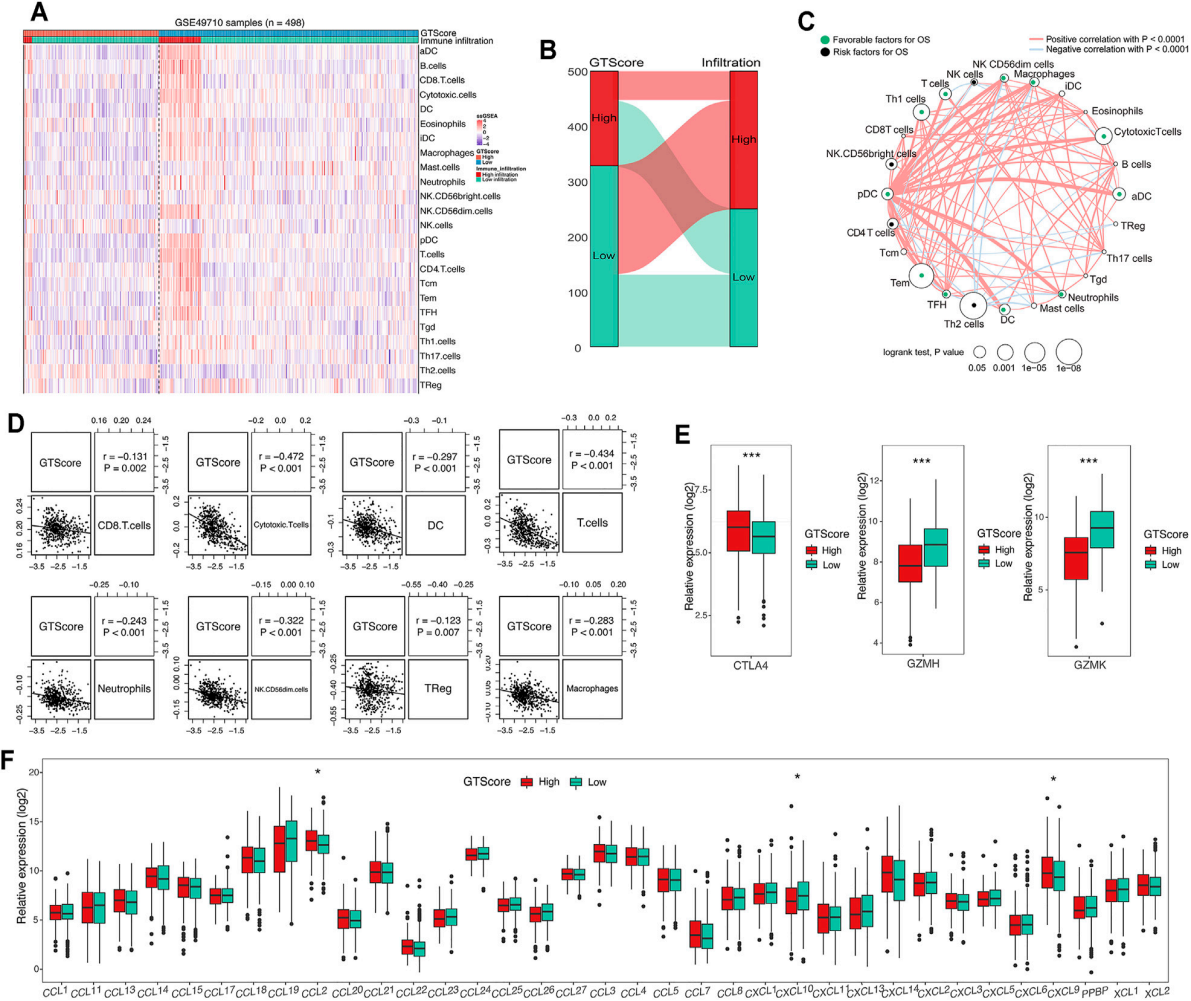
Correlation of GTscore with immune cell infiltration. **(A)** Heat map of the association between the risk score and the abundance of 24 immune cell types in NB. **(B)** Sankey plot showing the association between GTscore and immune infiltration. **(C)** Cellular interaction of the immune cell types in NB. The thickness of the line represents the strength of the correlation. Correlation between GTscore and immune cell infiltration **(D)**, immune checkpoint genes **(E)**, and the expression levels of chemokines **(F)**. ^*^ *p* < 0.05, ^***^ *p* < 0.001.

GTs are closely associated with tumor immune escape (Wang et al., 2019). The tumor microenvironment, which constitutes various immune cell subsets, affects the antitumor effects of immunotherapy (Liu and Joshi, 2020). Therefore, a difference in immune cell infiltration between the two groups was observed. To compare immune cell infiltration between separate GTscore groups, we utilized the ssGSEA approach to explore the relative abundance of immune cell types based on expression profiling data and curated immune gene signatures. Unsupervised hierarchical clustering was applied to categorize the cohort into two infiltration subgroups: a high infiltration group and a low infiltration group. We then investigated the association of immune cell infiltration levels and GTscore. The results showed that most of the 498 NB samples had a low degree of immune cell infiltration (Figure 7A), and the high GTscore group was related to low immune cell infiltration patients (*χ* ^2^ = 38.795, *p* < 0.001) (Figure 7B). The immune cell network showed that the content proportion of T-cell subgroups was significantly related to the OS of NB patients (*p* < 0.05) (Figure 7C). We found that the GTscore was negatively correlated with the expression levels of CD56^dim^ natural killer (NK) cells, CD8^+^ T cells, cytotoxic T cells, dendritic cells (DCs), neutrophils, and T cells but also negatively correlated with the levels of macrophages and regulatory T (Treg) cells (all *p* < 0.001) (Figure 7D). Furthermore, we found that the expression level of CTLA-4 was significantly positively correlated with the GTscore, whereas that of GZMH and GZMK was significantly negatively correlated with the GTscore (all *p* < 0.05) (Figure 7E). Considering the role of chemokines and immune checkpoints in immune cell infiltration and activation, we also investigated the association of their expression levels with the GTscore. As shown in Figure 7F, the chemokines CCL2, CXCL9, and CXCL10 were significantly different between the low and high GTscore groups.

### The Predictive Value of GTscore for GD2 Phenotypes and NSE Expression Levels in NB and Other Neuroendocrine Tumors

**FIGURE 8:**
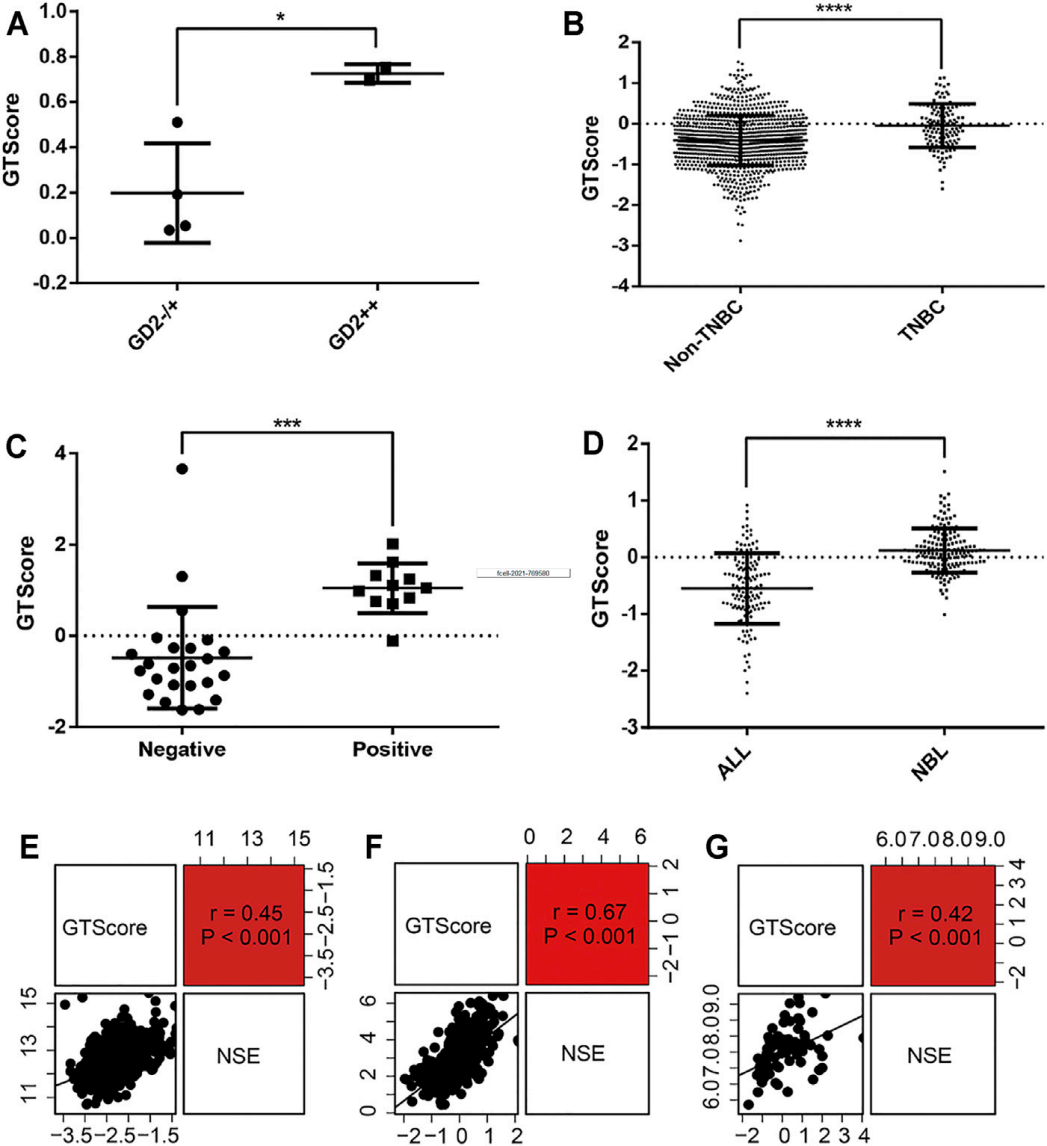
Correlation of GTscore with GD2 phenotype and NSE expression levels. **(A)** Differences in GTscore between the GD2 (−) HOS cell line, GD2 (+) U2OS cell line, U-373 cell line, SH-SY5Y cell line, and GD2 (++) T98G and IMR-32 cell lines. **(B)** Differences in GTscore between GD2 (−) non-TNBC and GD2 (+) TNBC samples. **(C)** Differences in GTscore between GD2 (+) and GD2 (−) NB samples. **(D)** Differences in GTscore between GD2 (−) ALL and GD2 (+) NB samples. Scatterplots showing the correlations between GTscore and NSE expression levels in NB **(E)**, melanoma **(F)**, and small cell lung cancer **(G)** samples. ^*^ *p* < 0.05, ^***^ *p* < 0.001, ^****^ *p* < 0.0001.

As GD2-positive tumors are more responsive to anti-GD2 targeted immunotherapy, it is imperative to detect the expression levels of GD2 in tumor cells. Therefore, we explored the relationship between GTscore and GD2 phenotypes. In the GSE142293 dataset, the authors analyzed GD2 status of distinct cancer cell lines, including GD2 (−) cell line HOS; GD2 (+) cell lines U2OS, U-373, SH-SY5Y; and GD2 (++) cell lines T98G, IMR-32. We calculated the GTscore for each cell line, and the results showed that the GTscore of the GD2 (++) cell lines was significantly higher than that of the GD2 (+) and GD2 (−) cell lines (*p* < 0.05) (Figure 8A). We analyzed the difference in GTscore between GD2 (−) ALL and GD2 (+) NB (TARGET database) and GD2 (−) non-TNBC and GD2 (+) TNBC samples (TCGA database). The results showed that GTscores were significantly higher in GD2 (+) samples, corresponding to the GD2 level (*p* < 0.05) (Figures 8B, D). Moreover, we analyzed the difference in GTscore between flow cytometry–identified 11 GD2 (+) and 24 GD2 (−) NB samples in datasets GSE90689 and GSE112447. The results also demonstrated that the GTscore was significantly higher in GD2 (+) samples (*p* < 0.05) (Figure 8C).

NSE is currently considered a useful marker in the diagnosis and prognosis of neuroendocrine tumors (Braga et al., 2013). Therefore, we investigated the relationship between GTscore and NSE expression levels in NB (GSE49710), melanoma (TCGA database), and small cell lung cancer (TCGA database). The results showed that the GTscore was positively correlated with the expression levels of NSE (all *p* < 0.001) (Figures 8E–G), suggesting that the GTscore could be used as a marker of neuroendocrine tumors.

### GTscore as a Predictive Indicator of Response to Immunotherapy of Neuroendocrine Tumors

ICI therapy has been used for cancer patients (Bagchi et al., 2021). Unfortunately, only a minority of patients can benefit from immunotherapy. As mentioned previously, the GTscore was negatively related to the degree of cytotoxic T-cell infiltration, which was reported to be related to the immunotherapeutic effects of NB patients (Figure 7C). Furthermore, we found that the GTscore was associated with GD2 and NSE, significant markers for neuroendocrine tumors. Because the ICI treatment dataset was not available in NB samples, we chose skin melanoma to investigate whether the GTscore could predict immunotherapeutic benefits in the ICI cohort of skin melanoma (TCGA-SKCM).

**FIGURE 9:**
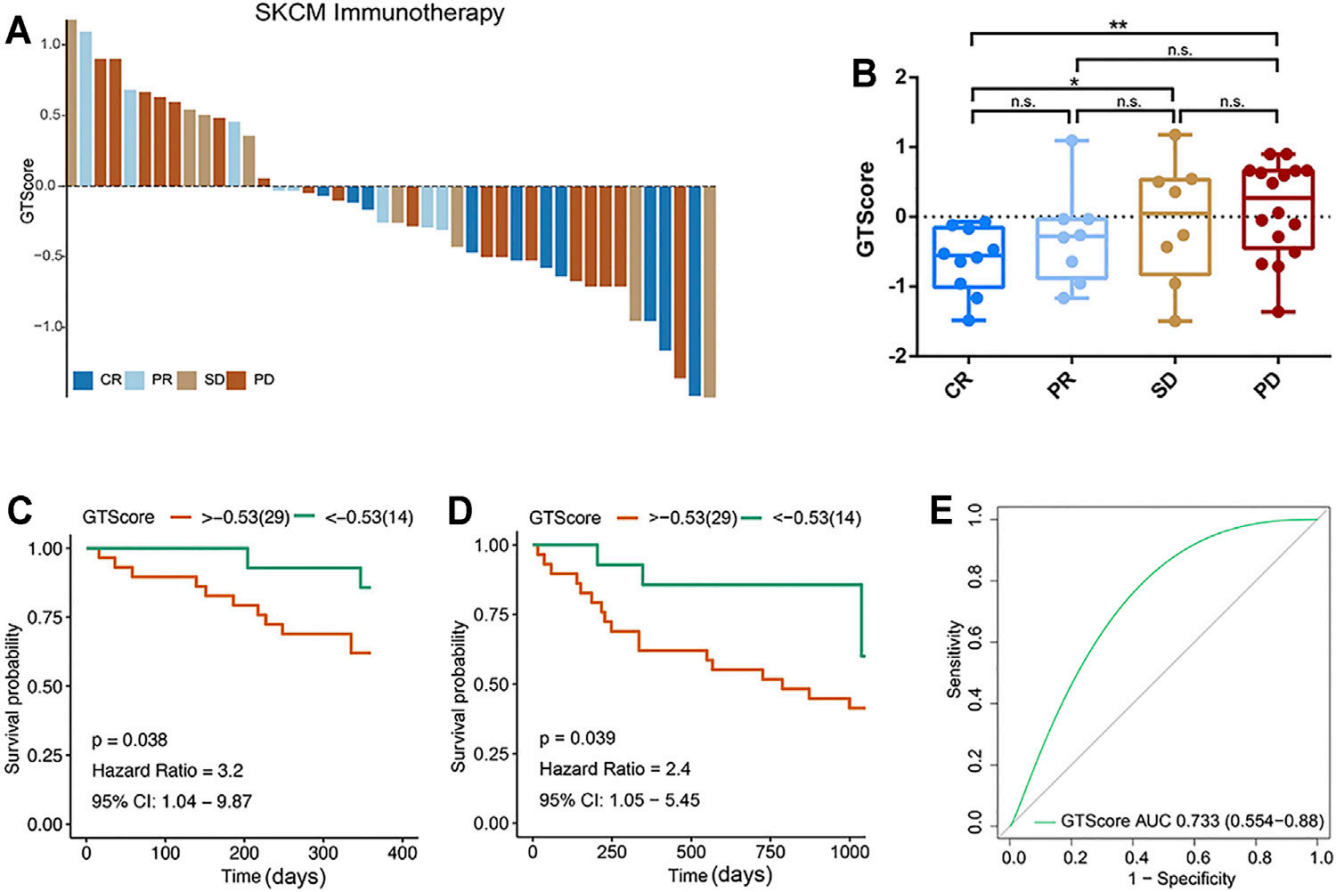
GTscore as a predictive indicator of the response to immunotherapy of melanoma. **(A)** Waterfall plot showing the GTscore and corresponding immunotherapy efficacy of individual patients. **(B)** Boxplot showing the distribution of GTscore in groups with different anti–PD-1 clinical response statuses. GTscore predicting the 1-year **(C)** and 3-year **(D)** OS rate of patients receiving anti–PD-1 immunotherapy *via* Kaplan–Meier curves. **(E)** The predictive value of GTscore in patients receiving anti–PD-1 immunotherapy *via* ROC curve analysis. ^*^ *p* < 0.05, ^**^ *p* < 0.01.

According to the evaluation criteria for the efficacy of solid tumors, the results showed that GTscore was negatively related to the clinical efficacy (Figure 9A), and the GTscore of CR samples was significantly lower than that of PD and SD samples (*p* < 0.05) (Figure 9B). The 1- and 3-year PFS of high GTscore patients was lower than that of low GTscore patients (HR = 3.20 [95% CI = 1.04–9.87, *p* = 0.038] and HR = 2.40 [CI = 1.05–5.45, *p* = 0.039], respectively) (Figures 9C, D). The AUC value of the GTscore predicting the benefits of anti–PD-1 immunotherapy was 0.733 (Figure 9E). These results suggested the authentic potency of the GTscore as a biomarker for immunotherapeutic benefit predictions and helped to develop novel treatment strategies for neuroendocrine tumors.

## Discussion

NB, the most common childhood extracranial solid tumor, has a poor prognosis, despite comprehensive treatment. Hence, it is necessary to explore the novel prognostic indicators to improve the prognosis of NB patients. Bioinformatics analysis methods provide a new idea for the evaluation of the prognosis of NB patients. A number of models to predict the prognosis of NB, including MYCN-related signatures (Wang et al., 2020), hypoxia gene signatures (Fardin et al., 2010), and immune-based models (Song et al., 2020), have been reported. A large number of studies have shown that GTs are related to cancer progression and can potentially serve as novel biomarkers (Potapenko et al., 2010; Burchell et al., 2018; Tang et al., 2019). GT-based signatures have been constructed to predict the survival in head and neck squamous cell carcinoma (Jin and Yang, 2019) and pancreatic cancer (Gupta et al., 2020). However, the association between GT-related signatures and the prognosis of patients with NB is still unknown. To our knowledge, this study is the first time to identify GTs associated with the prognosis of NB by bioinformatics methods.

In this study, we identified 64 GT DEGs associated with the progression and prognosis of NB patients in the GEO dataset (GSE49710). Moreover, these genes were enriched in the glycosphingolipid biosynthetic process. GD2 is an important glycosphingolipid that has been used as a target for NB immunotherapy (Suzuki and Cheung, 2015). Among them, 10 genes were associated with the GD2 synthesis pathway. Subsequently, based on 10 genes, we identified a GTscore including four GTs using LASSO, Cox regression, and SVM analysis. A novel 4-GT gene signature was then built. Each patient was assigned a score based on GTscore and then assigned to high and low GTscore groups by the optimal cutoff risk scores. In the training set, our GTscore showed a strong ability to predict the prognosis of NB. In addition, the prognostic power of the GTscore was verified in the validation set. Currently, clinicopathological factors are still the most important guidelines and are used to predict the prognosis of NB. Our studies revealed that our GTscore was closely associated with many malignant clinical features. Of note, we found that the GTscore was also an independent prognostic factor. Therefore, our GTscore could serve as a reliable prognostic indicator.

Several studies have shown that GD2 is an important cell-surface molecule involved in cancer biological processes, including tumor cell proliferation, metastasis, invasion, and signal transduction (Mujoo et al., 1987; Iwasawa et al., 2018; Ohmi et al., 2018). GD2 is synthesized from simpler ganglioside molecules by several GTs (Groux-Degroote et al., 2017). Our GTscore comprises four genes, including B4GALT5, B3GALT4, ST3GAL2, and ST3GAL3. Regrettably, few of them were identified as prognostic biomarkers in NB. However, there are still some studies involved in the function of these genes in other tumors. B4GALT5 is a member of the β1,4-galactosyltransferase family. It has been reported that B4GALT5 can suppress apoptosis and enhance cancer cell proliferation (Xu et al., 2002; Jiang and Gu, 2010). B4GALT5 can also mediate the stemness of breast cancer and is considered to be a prognostic biomarker for breast cancer (Tang et al., 2020). Chatterjee et al*.* reported that B4GALT5 might be a candidate diagnostic and therapeutic biomarker for colorectal cancer (Chatterjee et al., 2019). The B3GALT4 gene belongs to the β-1,3-galactosyltransferase (β3GalT) gene family. It was overexpressed in multiple malignant tumor tissues. Seko et al*.* had confirmed that B3GALT4 was a promising biomarker for the diagnosis of gynecological cancers (Seko et al., 2009). Zhang et al. also found that B3GALT4 was identified as a prognostic and therapeutic biomarker in colorectal cancer (Zhang et al., 2018). α2,3-Sialyltransferases ST3GAL2 and ST3GAL3 are responsible for sialyl-Lewis(x) synthesis, which is associated with cancer aggressiveness. It has been found that ST3GAL2 is a poor prognostic factor in breast cancer patients treated with chemotherapy (Aloia et al., 2015). Saito et al. also reported that ST3GAL2 activation enhanced the malignant progression of renal cell carcinoma (Saito et al., 2003). Mehta et al*.* identified that ST3GAL2 and ST3GAL3 overexpression was significantly associated with tumor stage, invasiveness, and metastasis of oral squamous cell carcinoma (Mehta et al., 2020). In pancreatic adenocarcinoma, overexpression of ST3GAL3 correlates with tumor progression (Perez-Garay et al., 2010). These studies supported our GTscore as a prognostic factor for NB patients.

Tumor microenvironment, including stromal cells and immune cells, is closely related to tumor progression in NB (Joshi, 2020). Tumor-infiltrating immune cells are related to disease progression, therapeutic responses, and prognosis (Luen et al., 2017; Ding et al., 2018; Lin et al., 2020). Cancer cells escape from immune surveillance through the imbalance of infiltrating immune cells in the tumor microenvironment (Vanichapol et al., 2018). Consequently, we investigated the variations in immune cell infiltration between the high and low GTscore groups. The results indicated that the numbers of CD8^+^ T cells, cytotoxic T cells, DCs, CD56^dim^ NK cells, and T cells were significantly lower in the high GTscore group, indicating that our signature may interfere with immune cell infiltration in NB. Gangliosides regulate T-cell activation by affecting the formation of the cell membrane signal complex (Sorice et al., 2004). It has been reported that inhibition of B4Galt5 and B4Galt6 results in higher invariant (i) NK T (iNKT) cell–activating capacities of adipocytes (Rakhshandehroo et al., 2019). It has been reported that overexpression of CD8^+^ T cells or CD56^dim^ NK cells is associated with favorable prognosis, whereas Treg cells are involved in an unfavorable prognosis (Johnson et al., 2007; Mina et al., 2015; Troschke-Meurer et al., 2019). However, contrary to previous research results, we found that the GTscore was negatively correlated with the levels of macrophages and Treg cells. Thus, further detailed analyses are required to assess the relationships between GTscore and immune cell infiltration. Chemokines play an important role in immune cell infiltration and immunotherapy. Interestingly, CCL2, CXCL9, and CXCL10 were significantly different between the low and high GTscore groups. It has been reported that CCL2 could mediate the migration of iNKT cells to NB *in vitro* (Metelitsa et al., 2004). It is generally believed that CXCL9 and CXCL10 play an important role in the recruitment and activation of T cells. Importantly, Bocca et al. found that CXCL10 could attract CXCR3^+^ GD2-CAR T cells *in vitro* (Bocca et al., 2017). In addition, several immune checkpoints including GZMH*,* GZMK, and CTLA-4 were assessed. Interestingly, the expression level of CTLA-4 was significantly positively correlated with the GTscore, whereas that of GZMH and GZMK was significantly negatively correlated with the GTscore. CTLA-4 is a receptor that suppresses the T-cell function (Chen and Flies, 2013). Both GZMH and GZMK play a critical role in driving NK cell cytotoxicity and memory CD8 T-cell differentiation (Fellows et al., 2007; Harari et al., 2009). These results may provide a useful reference for us to further explore the immune microenvironment of NB.

As GD2-positive tumors are more responsive to anti-GD2 immunotherapy, it is necessary to screen GD2 molecular phenotypes on tumor cells. Recently, Sorokin et al*.* built a gene expression signature to predict the GD2-positive tumor phenotypes (Sorokin et al., 2020). Here, we investigated the ability of the GTscore to distinguish the GD2-positive phenotype in the four cases. Our analysis demonstrated that our GTscore displays good predictive ability for a putative GD2-positive phenotype. This model is helpful to predict the GD2 phenotype in clinical specimens from cancer patients. NSE can be useful for the diagnosis, prognosis, staging, and treatment of related neuroendocrine tumors (Faias et al., 2021). We found that our GTscore was positively correlated with NSE expression levels, indicating that the GTscore we developed could be used as a marker of neuroendocrine tumors.

Recently, immunotherapy has provided a promising strategy for NB (Kholodenko et al., 2018). However, only 20% to 30% of high-risk NB patients could benefit from immunotherapy (Voeller and Sondel, 2019). The current problem faced by researchers is how to identify patients who respond to immunotherapy. Although some studies have been done in this respect, there is still a lack of reliable markers. In the current study, we investigated the ability of the GTscore to predict the response of skin melanoma patients to anti–PD-1 immunotherapy. Our results indicated that the GTscore could reliably predict the anti–PD-1 therapy effect. In addition, the survival time of the low GTscore group was longer than that of the high GTscore group. On the basis of previous studies (Li et al., 1995), we hypothesize that cancer cells in the high GTscore subtype secrete the immunosuppressive molecule sGD2 (Ladisch et al., 1987), which inhibits T-cell proliferation and therefore likely contributes to immunotherapy resistance. However, the ability of the GTscore to predict the anti-GD2 immunotherapeutic response needs further verification.

There are several deficiencies in our research. First, another independent NB cohort that underwent GT assays and received anti-GD2 therapy with follow-up data will be required to validate our observation. Second, more laboratory experiments are needed to verify the detailed biological mechanism of the identified genes.

## Conclusion

We constructed a GTscore, which could provide a favorable prognostic value in NB. Furthermore, we confirmed that the GTscore had a good predictive ability for putative GD2-positive molecular phenotype and NSE expression levels. Finally, the GTscore could accurately predict the efficacy of immunotherapy in melanoma. Nevertheless, large-scale, multicenter, and prospective studies are necessary to confirm our results.

## Data Availability

The datasets presented in this study can be found in online repositories. The names of the repository/repositories and accession number(s) can be found below: https://www.ncbi.nlm.nih.gov/geo/, GSE49710, GSE90689, GSE142293 https://ocg.cancer.gov/programs/target, TARGET https://portal.gdc.cancer.gov/, TCGA.
