# Supplementary material for: Identification of a Glycosyltransferase Signature for Predicting Prognosis and Immune Microenvironment in Neuroblastoma
Source: Front Cell Dev Biol. 2022 Jan 6;9:769580. doi: 10.3389/fcell.2021.769580 (PMC8773256; doi:10.3389/fcell.2021.769580)
Supplement: Supplementary file 1 [file DataSheet1.pdf]

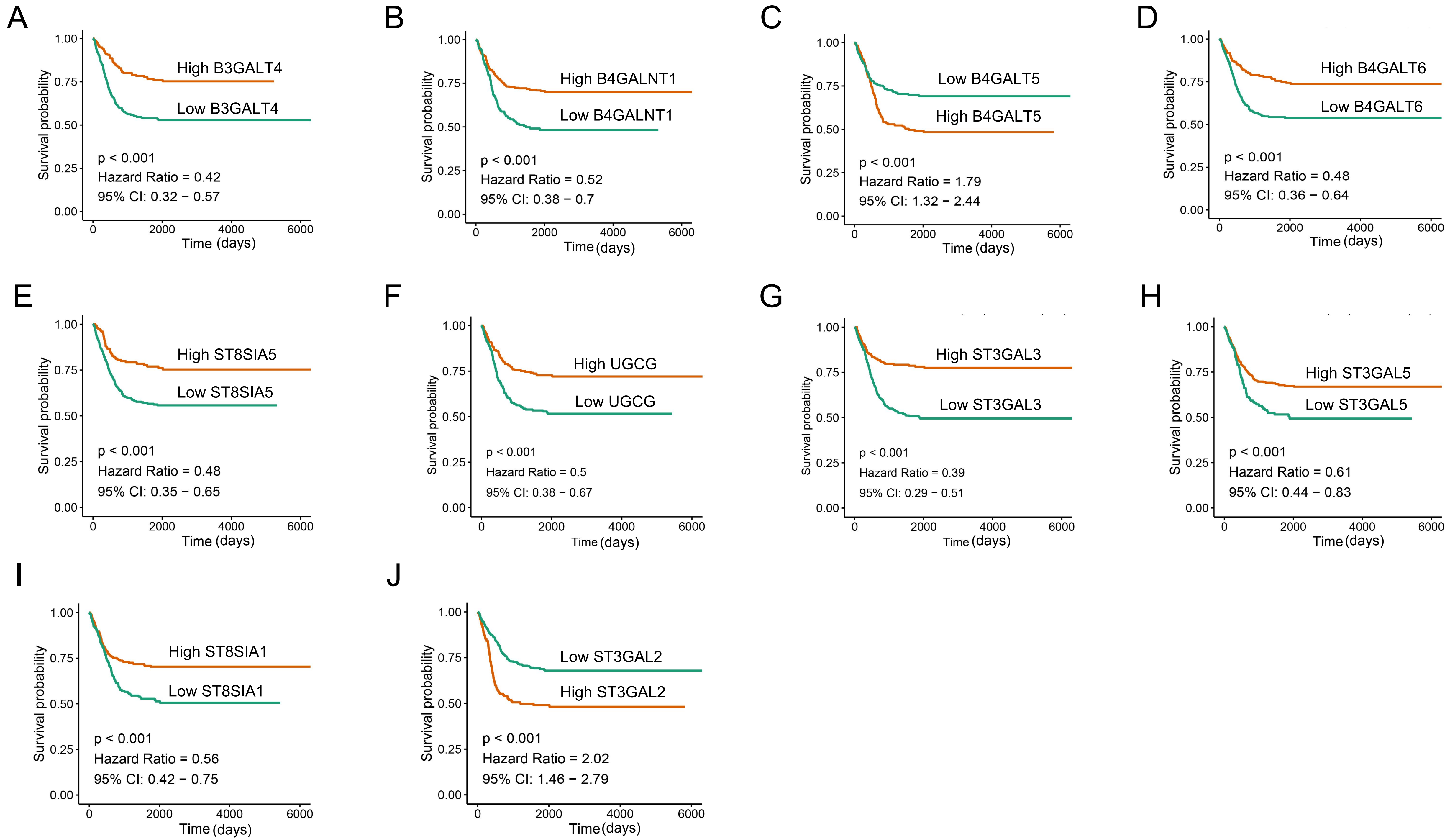
A
High B3GALT4
Low B3GALT4
p < 0.001
Hazard Ratio = 0.42
95% CI: 0.32 − 0.57
B
High B4GALNT1
Low B4GALNT1
p < 0.001
Hazard Ratio = 0.52
95% CI: 0.38 − 0.7
C
Low B4GALT5
High B4GALT5
p < 0.001
Hazard Ratio = 1.79
95% CI: 1.32 − 2.44
D
High B4GALT6
Low B4GALT6
p < 0.001
Hazard Ratio = 0.48
95% CI: 0.36 − 0.64
E
High ST8SIA5
Low ST8SIA5
p < 0.001
Hazard Ratio = 0.48
95% CI: 0.35 − 0.65
F
High UGCG
Low UGCG
p < 0.001
Hazard Ratio = 0.5
95% CI: 0.38 − 0.67
G
High ST3GAL3
Low ST3GAL3
p < 0.001
Hazard Ratio = 0.39
95% CI: 0.29 − 0.51
H
High ST3GAL5
Low ST3GAL5
p < 0.001
Hazard Ratio = 0.61
95% CI: 0.44 − 0.83
I
High ST8SIA1
Low ST8SIA1
p < 0.001
Hazard Ratio = 0.56
95% CI: 0.42 − 0.75
J
Low ST3GAL2
High ST3GAL2
p < 0.001
Hazard Ratio = 2.02
95% CI: 1.46 − 2.79
Survival probability
Time (days)

Supplementary FIGURE 1. Event-free survival analysis of 10 GD2-related GT genes in NB (based on GSE49710 dataset). (**A-J**) Expression levels of UGCG, B4GALT5, B4GALT6, B4GALNT1, B3GALT4, ST3GAL2, ST3GAL3, ST8SIA1, ST8SIA5 and ST3GAL5 are significantly related to the event-free survival of NB patients ($P < 0.05$).

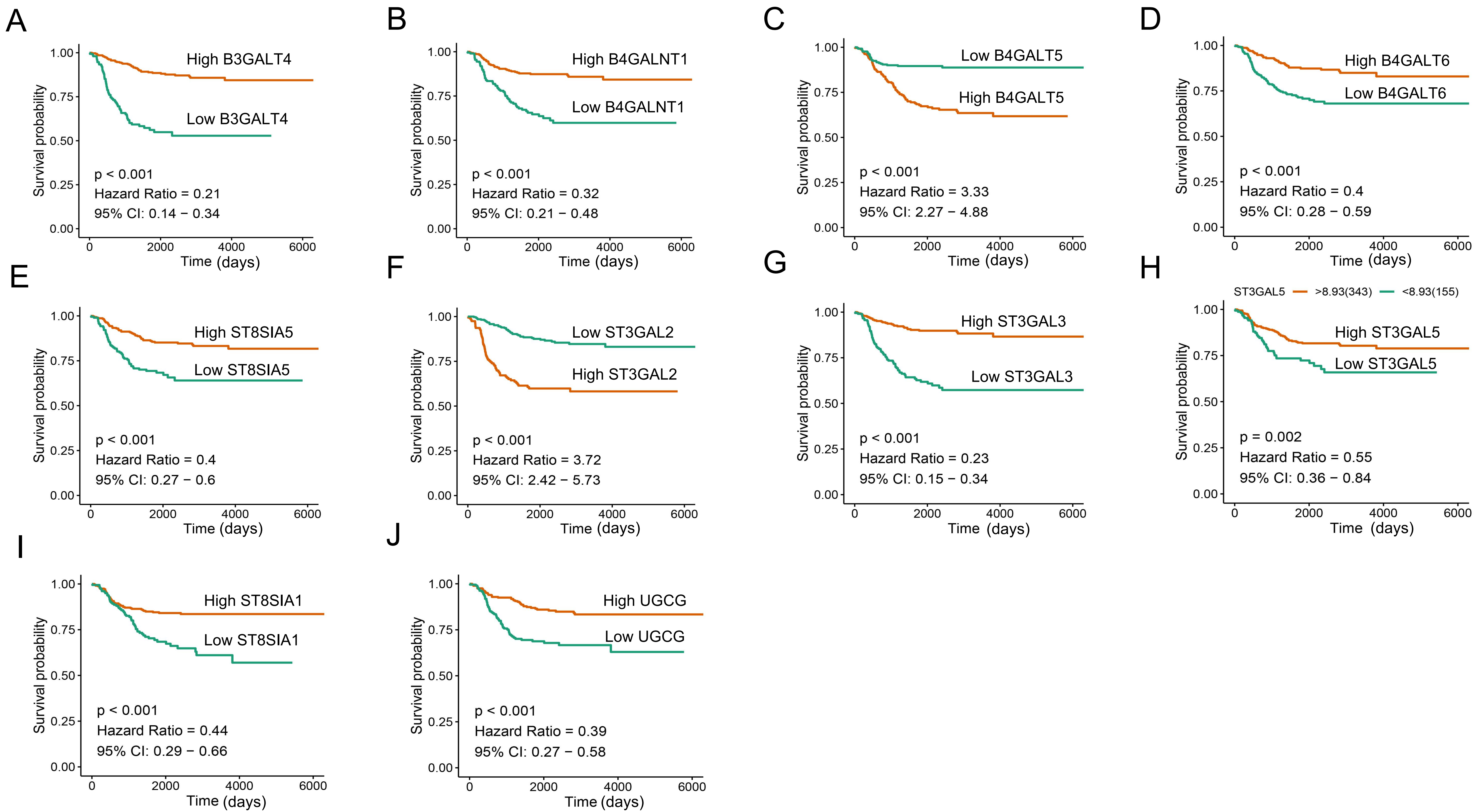
A
High B3GALT4
Low B3GALT4
p < 0.001
Hazard Ratio = 0.21
95% CI: 0.14 − 0.34
B
High B4GALNT1
Low B4GALNT1
p < 0.001
Hazard Ratio = 0.32
95% CI: 0.21 − 0.48
C
Low B4GALT5
High B4GALT5
p < 0.001
Hazard Ratio = 3.33
95% CI: 2.27 − 4.88
D
High B4GALT6
Low B4GALT6
p < 0.001
Hazard Ratio = 0.4
95% CI: 0.28 − 0.59
E
High ST8SIA5
Low ST8SIA5
p < 0.001
Hazard Ratio = 0.4
95% CI: 0.27 − 0.6
F
Low ST3GAL2
High ST3GAL2
p < 0.001
Hazard Ratio = 3.72
95% CI: 2.42 − 5.73
G
High ST3GAL3
Low ST3GAL3
p < 0.001
Hazard Ratio = 0.23
95% CI: 0.15 − 0.34
H
ST3GAL5 >8.93(343) <8.93(155)
High ST3GAL5
Low ST3GAL5
p = 0.002
Hazard Ratio = 0.55
95% CI: 0.36 − 0.84
I
High ST8SIA1
Low ST8SIA1
p < 0.001
Hazard Ratio = 0.44
95% CI: 0.29 − 0.66
J
High UGCG
Low UGCG
p < 0.001
Hazard Ratio = 0.39
95% CI: 0.27 − 0.58
Survival probability
Time (days)

Supplementary FIGURE 2. Overall survival analysis of 10 GD2-related GT genes in NB (based on GSE49710 dataset). (**A-J**) Expression levels of UGCG, B4GALT5, B4GALT6, B4GALNT1, B3GALT4, ST3GAL2, ST3GAL3, ST8SIA1, ST8SIA5 and ST3GAL5 are significantly related to the overall survival of NB patients ($P < 0.05$).

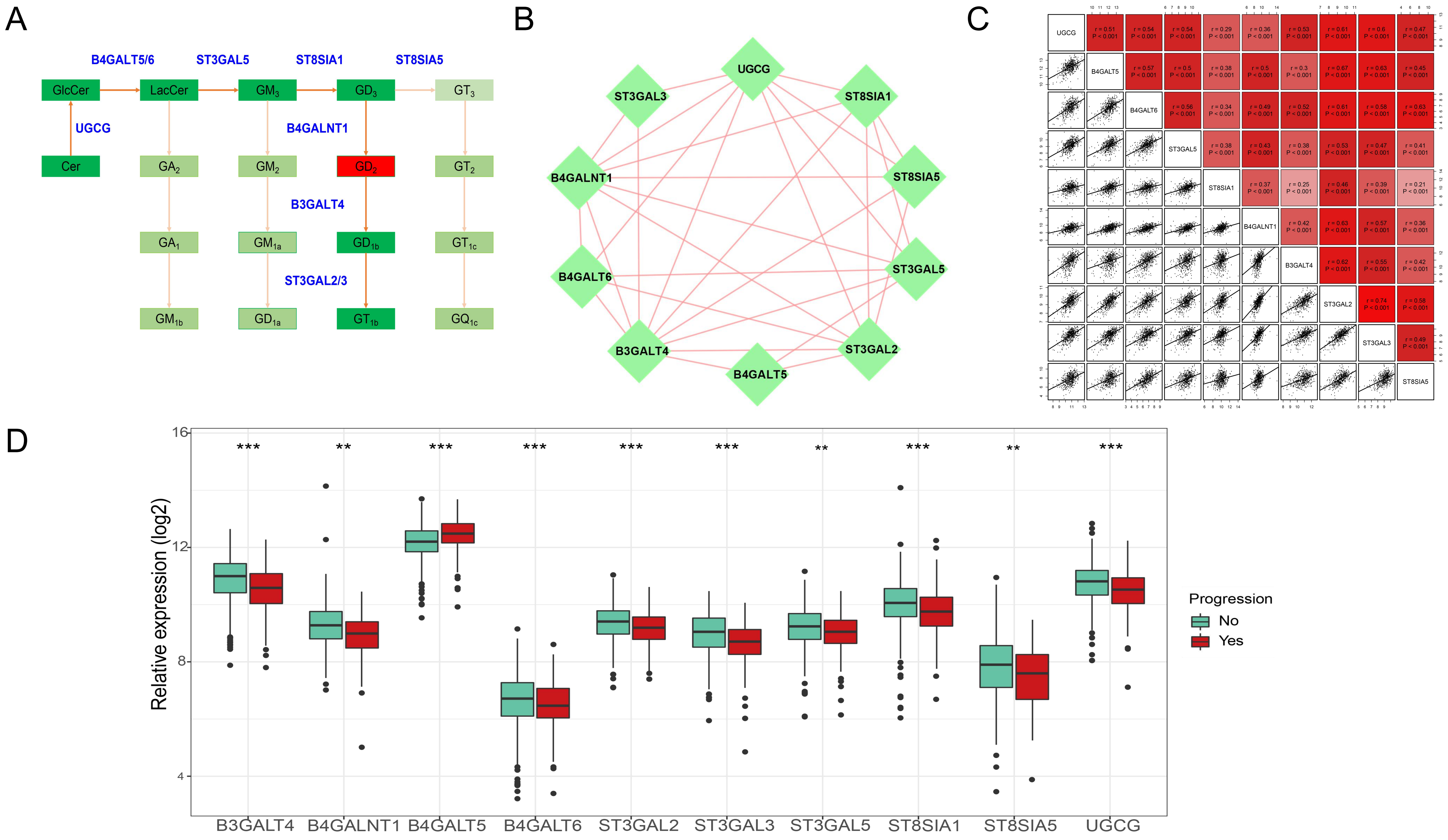
A
B4GALT5/6
ST3GAL5
ST8SIA1
ST8SIA5
GlcCer
LacCer
GM3
GD3
GT3
UGCG
B4GALNT1
Cer
GA2
GM2
GD2
GT2
B3GALT4
GA1
GM1a
GD1b
GT1c
ST3GAL2/3
GM1b
GD1a
GT1b
GQ1c
B
UGCG
ST3GAL3
ST8SIA1
B4GALNT1
ST8SIA5
B4GALT6
ST3GAL5
B3GALT4
ST3GAL2
B4GALT5
C
D
Relative expression (log2)
B3GALT4
B4GALNT1
B4GALT5
B4GALT6
ST3GAL2
ST3GAL3
ST3GAL5
ST8SIA1
ST8SIA5
UGCG
Progression
No
Yes

Supplementary FIGURE 3. The interaction between GD2-related GT genes. (**A**) Summary of the synthetic pathway for ganglioside GD2 mediated by glycosyltransferases. (**B**) Protein-Protein Interaction (PPI) network of 10 GD2-related GT genes constructed in STRING. (**C**) Scatterplots showing GD2-related GT genes were highly inter-correlated. (**D**) The expression of 10 GD2-related GT genes between progression and non-progression NB samples.The upper and lower ends of the boxes represented interquartile range of values. The lines in the boxes represented median value, and black dots showed outliers. The asterisks represented the statistical p value (** $P < 0.01$; *** $P < 0.001$).

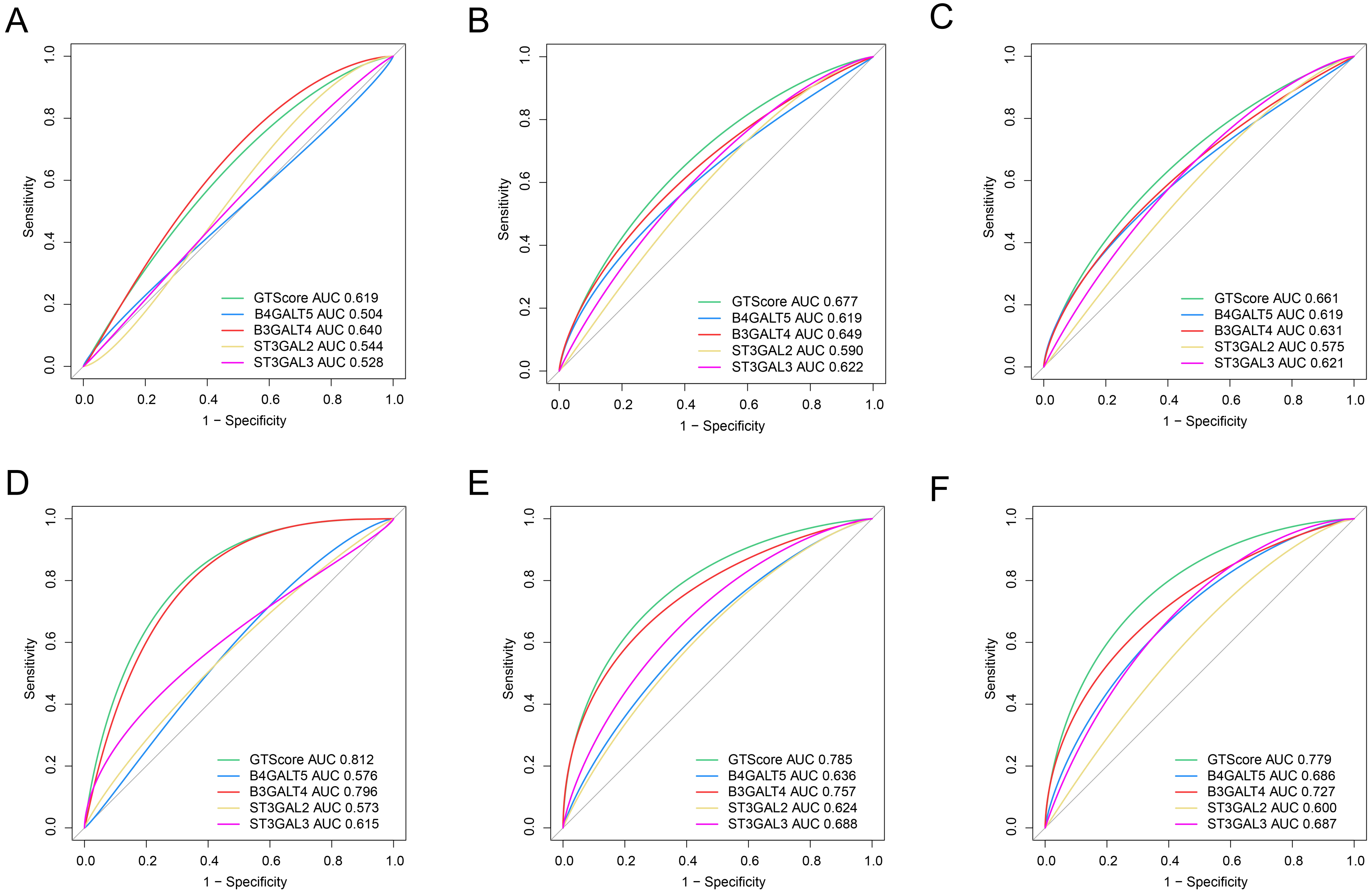
A
GTScore AUC 0.619
B4GALT5 AUC 0.504
B3GALT4 AUC 0.640
ST3GAL2 AUC 0.544
ST3GAL3 AUC 0.528
B
GTScore AUC 0.677
B4GALT5 AUC 0.619
B3GALT4 AUC 0.649
ST3GAL2 AUC 0.590
ST3GAL3 AUC 0.622
C
GTScore AUC 0.661
B4GALT5 AUC 0.619
B3GALT4 AUC 0.631
ST3GAL2 AUC 0.575
ST3GAL3 AUC 0.621
D
GTScore AUC 0.812
B4GALT5 AUC 0.576
B3GALT4 AUC 0.796
ST3GAL2 AUC 0.573
ST3GAL3 AUC 0.615
E
GTScore AUC 0.785
B4GALT5 AUC 0.636
B3GALT4 AUC 0.757
ST3GAL2 AUC 0.624
ST3GAL3 AUC 0.688
F
GTScore AUC 0.779
B4GALT5 AUC 0.686
B3GALT4 AUC 0.727
ST3GAL2 AUC 0.600
ST3GAL3 AUC 0.687
Sensitivity
1 − Specificity

Supplementary FIGURE 4. The prognostic value of GTscore is superior to the individual gene. Receiver operating characteristic (ROC) analysis for 1-(**A**), 3-(**B**), 5-(**C**) year EFS prediction in NB patients. ROC analysis for 1-(**D**), 3-(**E**), 5-(**F**) year OS prediction in NB patients. The Area Under Curve (AUC) were used to assess the prognostic accuracy.

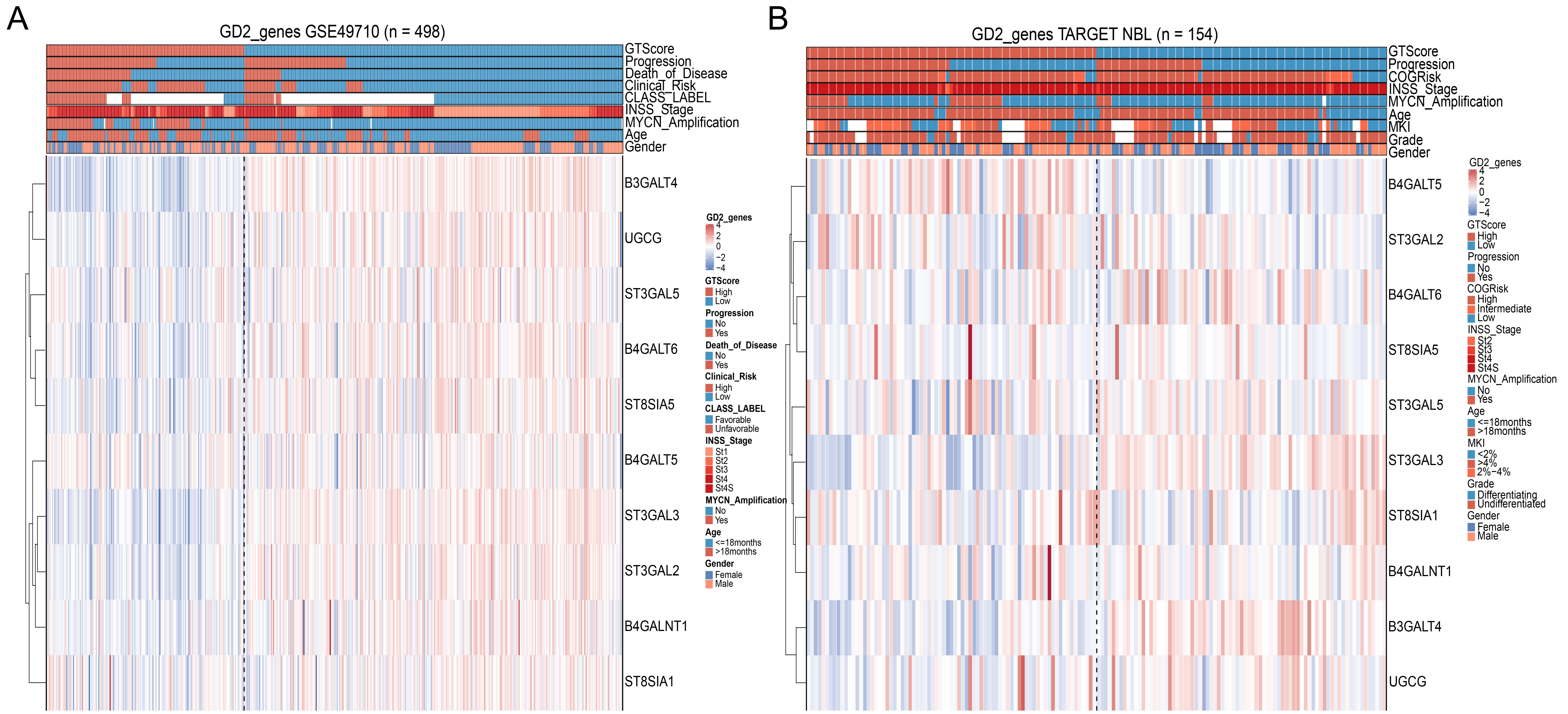

A
GD2_genes GSE49710 (n = 498)
GTScore
Progression
Death_of_Disease
Clinical_Risk
CLASS_LABEL
INSS_Stage
MYCN_Amplification
Age
Gender
B3GALT4
UGCG
ST3GAL5
B4GALT6
ST8SIA5
B4GALT5
ST3GAL3
ST3GAL2
B4GALNT1
ST8SIA1
GD2_genes
4
2
0
−2
−4
GTScore
High
Low
Progression
No
Yes
Death_of_Disease
No
Yes
Clinical_Risk
High
Low
CLASS_LABEL
Favorable
Unfavorable
INSS_Stage
St1
St2
St3
St4
St4S
MYCN_Amplification
No
Yes
Age
<=18months
>18months
Gender
Female
Male
B
GD2_genes TARGET NBL (n = 154)
GTScore
Progression
COGRisk
INSS_Stage
MYCN_Amplification
Age
MKI
Grade
Gender
B4GALT5
ST3GAL2
B4GALT6
ST8SIA5
ST3GAL5
ST3GAL3
ST8SIA1
B4GALNT1
B3GALT4
UGCG
GD2_genes
4
2
0
−2
−4
GTScore
High
Low
Progression
No
Yes
COGRisk
High
Intermediate
Low
INSS_Stage
St2
St3
St4
St4S
MYCN_Amplification
No
Yes
Age
<=18months
>18months
MKI
<2%
>4%
2%~4%
Grade
Differentiating
Undifferentiated
Gender
Female
Male

Supplementary FIGURE 5. Heatmap showing the correlation of GTscore and clinical characteristics. (**A**) The training set. (**B**) The validating set.
